# A randomised controlled pilot trial of the influence of non-native English accents on examiners’ scores in OSCEs

**DOI:** 10.1186/s12909-020-02198-y

**Published:** 2020-08-15

**Authors:** An Kozato, Nimesh Patel, Kiyoshi Shikino

**Affiliations:** 1 Queen Mary University of London, Centre for Medical Education, Institute of Health Sciences Education, Barts and The London School of Medicine & Dentistry, Garrod Building, Turner Street, London, UK; 2 Department of General Medicine, Chiba University Hospital, 1-8-1, Inohana, Chuo-ku, Chiba-city, Chiba, Japan

**Keywords:** OSCE, Stereotypes, Bias, Language, Accent, Examiner, Clinical competence

## Abstract

**Background:**

Objective structured clinical examinations (OSCEs) are important aspects of assessment in medical education. There is anecdotal evidence suggesting that students with non-native English accents (NNEA) may be subjected to unconscious bias. It is imperative to minimise the examiners’ bias so that the difference in the scores reflects students’ clinical competence. Research shows NNEAs can cause stereotyping, often leading to the speaker being negatively judged. However, no medical education study has looked at the influence of NNEAs in assessment.

**Methods:**

This is a randomized, single-blinded controlled trial. Four videos of one mock OSCE station were produced. A professional actor played a medical student. Two near identical scripts were prepared. Two videos showed the actor speaking with an Indian accent and two videos showed the actor speaking without the accent in either script. Forty-two OSCE examiners in the United Kingdom (UK) were recruited and randomly assigned to two groups. They watched two videos online, each with either script, each with a different script. One video with a NNEA and one video was without. Checklist item scores were analysed with descriptive statistics and non-parametric independent samples median test. Global scores were analysed with descriptive statistics and Mann-Whitney test.

**Results:**

Thirty-two examiners completed the study. The average scores for the checklist items (41.6 points) did not change when the accent variable was changed. Independent samples median test showed no statistically significant relationship between the accent and the scores (*p* = 0.787). For the global scores received by the videos with the NNEA, there were one less ‘Good’ grade and one more ‘Fail’ grade compared to those without the NNEA. Mann-Whitney test on global score showed lower scores for videos with NNEA (*p* = 0.661).

**Conclusions:**

Examiners were not biased either positively or negatively towards NNEAs when providing checklist or global scores. Further study is required to validate the findings of this study. More discussion is warranted to consider how the accent should be considered in current medical education assessment.

**Registration:**

Trial registration completed trial, ID: ISRCTN17360102, Retrospectively registered on 15/04/2020.

## Background

Objective structured clinical examinations (OSCEs) are assessment tools commonly used for assessing clinical competence [1, 2]. At an OSCE, medical students rotate through a set number of stations. In one station, there is typically one examiner who observes the student. There may be a simulated/real patient, a manikin, relevant equipment or clinical information. Each time a student enters a station, they are given a scenario with a task to complete, including practical procedures or patient managements. Students’ performance is marked by the examiner using a checklist or rating scale [3, 4]. It is reported OSCEs are superior to other assessment methods such as written examination or long cases [3] due to the high construct validity, the standardised cases and marking schemes.

It is important to minimise the examiner bias in OSCEs to accurately reflect performance differences. For this reason, formal examiner training is required [5, 6]. Under the Equality Act 2010, all students should be assessed independent of their protected characteristics. Therefore, equality, diversity and unconscious bias training is an integral part of the training scheme [5]. The training aim to ensure fair evaluation of diverse student population with use of case discussions and presentations.

Despite the implementation of examiner training, a systematic review has described that OSCEs were variable in their reliability scores [7], especially in communication skills stations where judgment on the listening skills and cultural competencies tended to be subjective. As the examiner’s measure for the communication is shaped by their cultural background [8], the outcome is likely to be idiosyncratic to the individual.

There have been anecdotes that students with non-native English accents (NNEA) were disadvantaged in an OSCE. While no research has been carried out to establish this issue, studies consistently show students’ performances vary depending on their protected characteristics such as gender and ethnicity [9–11]. It could be deduced that when examiners make judgements, the decision-making process is influenced by such characteristics and associated social identities.

Studies have consistently shown the correlation between the students’ language status and their performances in OSCE [11–14]. Acculturation was proposed to influence the communication skills in OSCE. Huhn et al. found that international medical students performed worse in the conversational skills stations [15].

Although no global consensus exists, NNEAs can be defined as the pronunciation of English speech, perceived to be produced by non-native speakers. Outside of medical education, perceptions towards NNEAs have been extensively investigated in speech and psychological studies [16]. It was reported the listeners with little experience in the non-native speakers’ first language correctly identified the foreign accents in the speeches presented [17]. The variation in consonants, vowels, speeds and speech timings have been identified as playing a part in the detection of NNEAs [18].

Various studies have demonstrated a change in attitudes exist towards speakers with NNEAs compared with native speakers. A speaker with a NNEA was judged less intelligible and comprehensible than a speaker with a NEA when the message delivered was the same [19]. The non-native accent was shown to be generally disfavoured by the listeners [20]. This bias in perception may lead to non-native speakers to be disadvantaged in an employment process [21]. Carlson and McHenry while using human resource professionals have shown that when the degree of perceived ‘accentedness’ of the respective employees were higher, the average employability score decreased [22].

Overall, there is a large body of evidence on the effects of NNEAs in assessments. However, the context of previous studies on accent bias could be different to that of an OSCE as examiners are instructed to assess based purely on the students’ clinical competencies. Although bias against foreign students has been demonstrated previously in medical education [23], the influence of the accent has never been considered. Further investigation is warranted to establish the role of NNEAs in medical education.

## Methods

### Study design, participant recruitment and ethical approval

A single-blind randomised, controlled study was performed to examine a relationship between NNEAs and OSCE scores. The hypothesis was that OSCE examiners scored students with NNEAs lower compared to students with NEAs when the performance is constant.

OSCE examiners in the UK were invited to participate in the study via email. Interested individuals contacted the researcher directly to receive the Participants Information Sheet and sign the Consent Form electronically. They were required to have completed formal examiner training. A sample size of 100 participants was set as the aimed sample size. Participants were randomly allocated to one of two groups. Group number were alternately allocated in the order to which consent forms were received. The recruitment and randomization process were conducted by the first author.

For the purpose of this study, revealing the true objective of the experiment was considered to predispose the examiners to bias. Therefore, they were simply informed the study evaluated “the assessment reliability in the current OSCE “and that they would be asked to assess two recorded performances of a student in an OSCE station. They were assured that further details would be provided once they completed the study. After completing the task, they were informed the study focused on the effect of NNEAs on OSCE examiners.

Participants were not offered any incentive to complete the study. They were informed they could withdraw from the study at any point. This study was approved by the Queen Mary Ethics of Research Committee (reference code QMERC2018/95).

This study followed the Consolidated Standards of Reporting Trials (CONSORT) reporting guideline (Supplement 1).

### Experiment materials and procedures

**Fig. 1:**
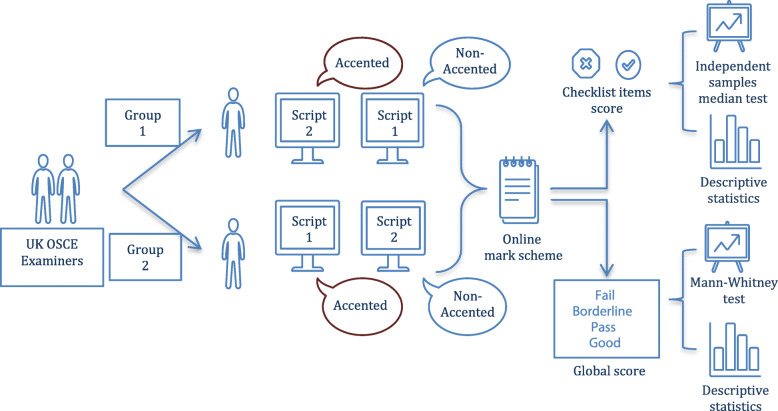
Overall experiment procedure. UK OSCE examiners were randomly allocated into two groups. They watched two OSCE videos with and without the NNEA. The scores were collected online and statistically analysed

As shown in Fig. 1, each group watched scripts 1 and 2 either with or without the NNEA. The scripts were based on a mock five-minute OSCE scenario which was a history taking station of leg pain, originally produced for training purposes. Both scripts contained identical contents, but the order of the student’s questions was changed. The purpose of this design was to mask the independent variable in the experiment from the participants. The crossover of scripts between the participant groups served to counteract the effect of the script variation.

The mark scheme included checklist items and a global score. This was considered appropriate as the rigid score scale allowed data quantification for the analysis. The competency level equivalent to a 3rd to 5th year clinical student was expected to pass the station.

The videos were filmed in a university recording booth with a built-in camera and audio recorder. The audio and image quality were checked by the first and second author before their distribution. The medical student role was played by a professional actor so that the accent variable could be manipulated. The actor was female and experienced in performing with and without a NNEA. In this case, an Indian accent was chosen as this corresponded to her ethnicity. The actor was instructed to control everything else including her body language, facial expression, voice tone and speed. The level of control was checked by the first and second author. Due to the resource limitation, only one actor was employed. The simulated patient was played by a student volunteer.

**Fig. 2:**
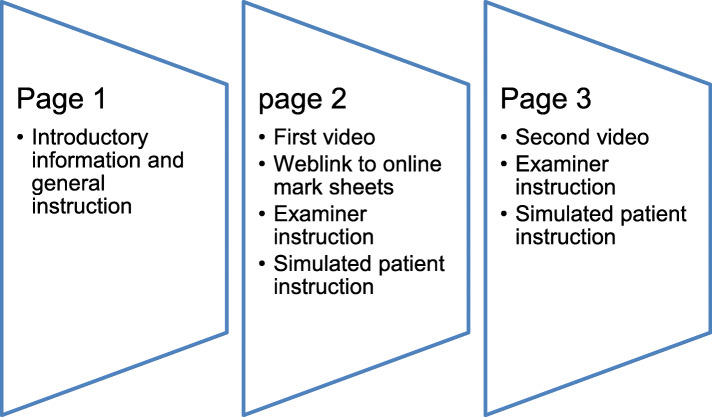
Summary of the online platform pages. The online platform pages for the experiment consisted of 3 pages. These included an introductory page and two pages with the video of OSCE stations, mark sheet weblinks and background information of the station

Two separate websites were created for the two participant groups and were used as the platforms for viewing the videos and accessing the mark sheet. Websites were created on Queen Mary Plus (QMPlus), Queen Mary’s online learning environment used for accessing learning materials based on Moodle. Each website contained three pages. Page 1 contained introductory information. Page 2 and 3 contained two videos with the examiner and simulated patient instruction and a weblink to online mark sheets (Fig. 2). The mark sheet was produced online using the Wufoo platfrom, a digital service for survey production. It contained twenty-one checklist items for which participants selected either “good”, “adequate” or “inadequate/not done”. At the end of the mark sheet, they were asked to provide a global score of “good”, “pass”, “borderline” or “fail”. The original content of the mark sheet was used in the study to ensure the validity of the result. Another form for demographic information was produced. Each participant was asked to fill in two identical mark sheets and one demographic information survey. All data was collected online upon submission, after which participants were notified of the true objective of the study. Participants were given contact information of the first author to report possible technical issues during the study. The data collection was conducted between 27th March 2019 to 30th April 2019.

### Analysis

Participants’ demographic data was analysed to determine if there were any differences between the groups. Secondly, the checklist items and the global ratings were analysed separately. Both analyses focused on the differences in scores between videos with and without NNEA. Therefore, the scores from the 2 accented videos and 2 non-accented videos were treated as a single dataset. The analysis was carried out using Microsoft Excel and SPSS Subscription version (Build 1.0.0.1327). Inter-rater reliability was also calculated using Fleiss Kappa analysis on SPSS. Effect size and statistical power analysis was performed on GPower version 3.1.

The grades for the checklist items were converted into numerical scores for the purpose of the analysis. The ordinal grades of ‘Good’, ‘Adequate’ and ‘Inadequate/Not done’ were converted into 3, 2 and 1 respectively. All twenty-one numerically converted grades in each marking were summed up. The checklist item score sums, referred as ‘checklist sum’ below, were treated as one variable in the analysis. For the checklist sums given to videos with and without the NNEA, the mean values were calculated and compared. The difference between the mean values were analysed with independent samples t-test. The dispersion of the checklist sums was visualised using box plots and compared using standard deviation. The individual examiner’s change in the checklist sum from the first to the second video was visualised by a line chart for each group. This was carried out to see whether there was a pattern in how the examiners scored the first and second videos. To identify relationship between the NNEA and checklist sums, non-parametric independent samples median test was performed.

The global scores were given in a categorical form in which examiners chose either ‘Good’, ‘Pass’, ‘Borderline’ or ‘Fail’. First, the number of each global score categories given to the videos with and without NNEA was counted. This was visualised using a bar chart. The individual examiner’s change in the global score from the first to the second video was visualised by a line chart. This was carried out to see whether there was a pattern in how the examiners scored the first and second videos. To identify a relationship between the NNEA and the global score, Mann-Whitney test was performed.

## Results

OSCE examiners in the UK were recruited between 27th March 2019 to 10th April 2019. Forty-two examiners were recruited and randomly assigned to group 1 or 2. Thirty-two examiners completed the study. There were fifteen and seventeen examiners in group 1 and 2, respectively. Nine examiners did not complete the study but the reasons were not given. One examiner was excluded from the analysis as the inclusion criteria were not met. None withdrew from the study after data submission. Three examiners marked the first video they watched twice in error. For these responses, the first responses were used for the analysis since the second response could be biased due to the previous knowledge of the video contents. The demographic data of the examiners are summarised in Table 1. The overall makeup of the examiners in each group were similar. Although most examiners received equality and diversity training, about half of the examiners in each group received unconscious bias trainings. Power analysis showed that total sample size of 148 was required for the statistical power of 0.8 for a moderate effect. Fleiss Kappa analysis showed there was a fair inter rater reliability for both accented videos, k = 0.313 (95% CI, 0.301 to 0.326) and non-accented videos, k = 0.310 (95% CI, 0.298 to 0.322).

**Table 1:** Participant demographics

*Characteristic*	*Group 1*	*Group 2*
	Frequency (%)	
Received unconscious bias training?		
Yes	9 (60)	9 (52)
No	6 (40)	8 (48)
Received equality and diversity training?		
Yes	14 (93)	14 (82)
No	1 (7)	3 (18)
Gender		
Male	12 (80)	11 (64)
Female	3 (20)	6 (36)
Ethnicity		
White	11 (73)	12 (70)
Asian	3 (20)	4 (24)
Black	0 (0)	0 (0)
Other	1 (7)	1 (6)
Level of training		
FY1	0 (0)	0 (0)
FY2	0 (0)	0 (0)
Core Training	0 (0)	0 (0)
Specialty training	1 (7)	1 (6)
Consultant	11 (73)	11 (65)
Other	3 (20)	5 (29)
Years of experience as an OSCE examiner		
< 5	7 (47)	8 (47)
5–10	6 (40)	5 (29)
11–15	2 (13)	3 (18)
16–20	0 (0)	1 (6)

Legend: The demographic information of the participants in each group was summarised

**Fig. 3:**
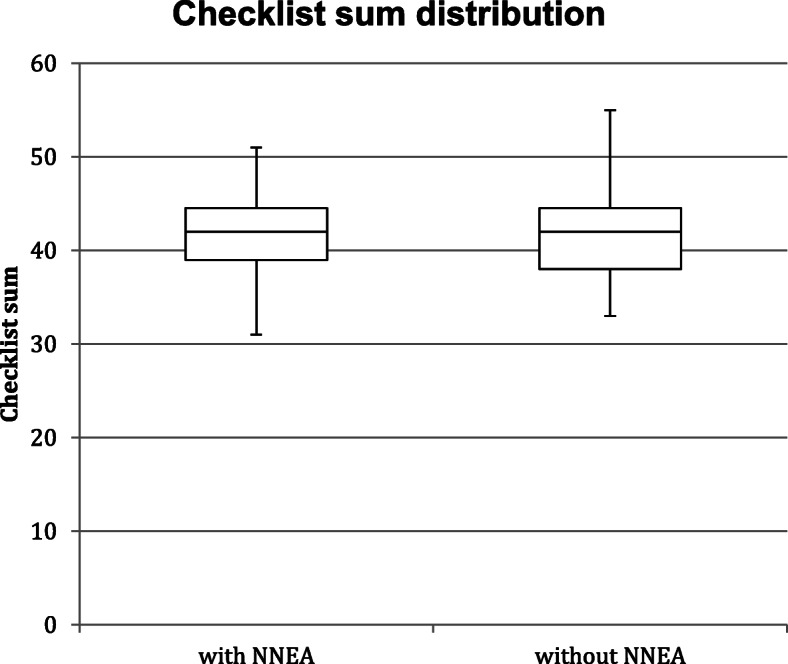
Checklist sum dispersion for videos with and without the NNEA. Checklist sum given to videos with the NNEA (Q1 = 39, Median = 42, Q3 = 44.5, Max = 51, Mi*n* = 31) (Left). Checklist sum given to videos without the NNEA (Q1 = 38, Median = 42, Q3 = 44.5, Max = 55, Min = 33) (Right)

The mean value of the checklist sums for videos with and without the NNEA were 41.6 ± Standard Deviation (SD) points v 41.6 ± SD, respectively (Table 2). Independent samples t-test for equality of means showed there was no statistically significant difference in the mean scores (*p* = 0.982). The dispersion of the scores was visualised using box plots (Fig. 3).

**Table 2:** Mean and standard deviation values for checklist sums

*Checklist sum*			
*With NNEA (n = 31)*		***Without NNEA (n = 31)***	
*Mean*	***Standard deviation***	***Mean***	***Standard deviation***
41.6	5.3	41.6	5.8

Legend: The mean values for the checklist sum were 41.6 for performances with and without the NNEA with the standard deviations of 5.3 and 5.8 respectively

The interquartile ranges for both video types were similar. However, the checklist sums for videos without the NNEA were more positively skewed than videos with the NNEA.

**Fig. 4:**
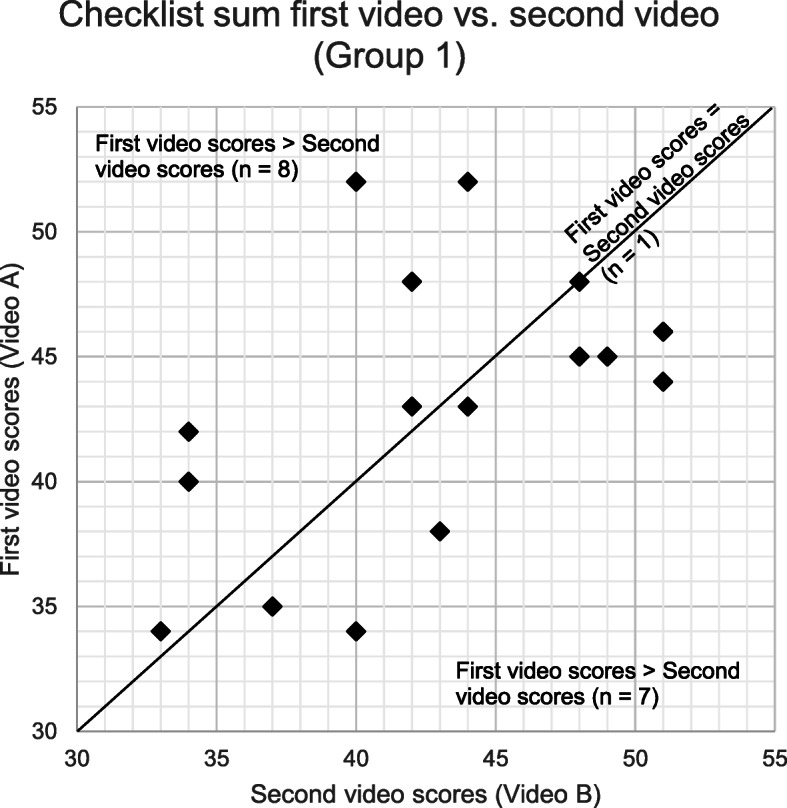
Checklist sum scores for group 1 – First video vs. second video. The figure shows the changes in checklist sums given to video A (NNEA present, script 2) and video B (NNEA absent, script 1) in group 1 (*n* = 16). Each point represent individual examiner

**Fig. 5:**
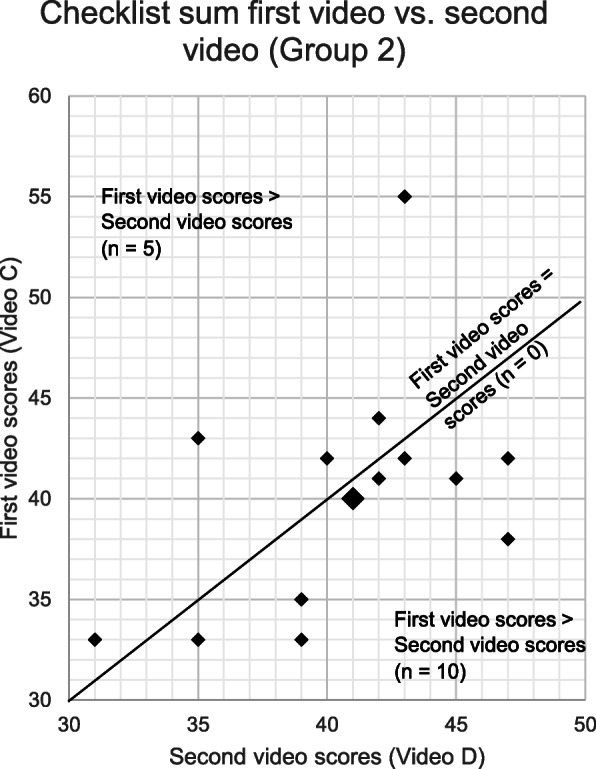
Checklist sum scores for group 2 – First video vs. second video. The figure shows the changes in checklist sums given to video C (NNEA present, script 1) and video D (NNEA absent, script 2) in group 2 (*n* = 15). A smaller point represent one examiner and the larger point represents two examiners

The change in the scores given to the first video to the second video was visualised for group 1 (Fig. 4) and group 2 (Fig. 5). Table 3 shows the variations in the pattern in which the checklist sums changed for group 1 and group 2.). More than 50% of the examiners in group 1 scored the first video A higher than the second video B. Only 33% of the examiners in group 2 scored the first video C higher than the second video D. Non-parametric independent samples median test was performed to analyse the relationship between the accent and checklist sums. The analysis showed there was no statistically significant effect of the accent variable on checklist sums (*p* = 0.787). An additional analysis on individual checklist items was performed and showed no statistically significant difference for all items.

**Table 3:** Pattern of score changes from the first video to second video in group 1 and 2

	*Change in score from the first video to the second video*					
	***Group 1, Video A to video B (n = 16)***			***Group 2, Video C to video D (n = 15)***		
	Increase	Decrease	Unchanged	Increase	Decrease	Unchanged
Number of examiners	7	8	1	5	10	0
Range of the score change	1–12	1–7	0	2–12	1–9	–

Legend: In group 1, 7 examiners rated the second video higher (range 1–12 points). 8 examiners rated the second video lower (range 1–7 points). 1 examiner gave same score to both videos. In group 2, 5 examiners rated the second video higher (range 2–12 points). 10 examiners rated the second video lower (range 1–9 points)

**Fig. 6:**
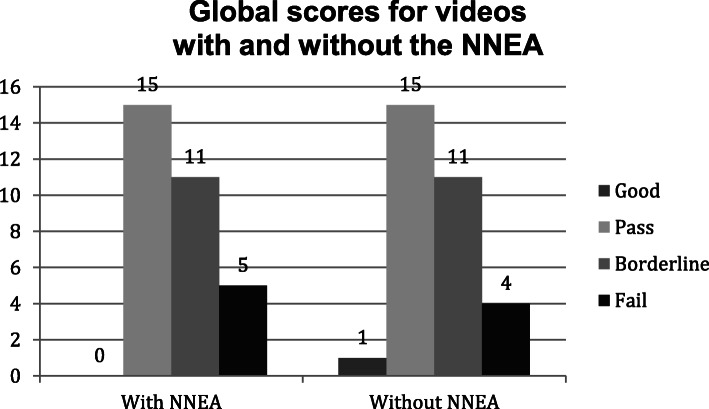
Bar charts showing global scores for videos with and without the NNEA. The bar chart shows the number of global scores given to videos with the NNEA (Left) and the number of global scores given to videos without the NNEA (Right). The counts for grade groups are shown on top of each bar

**Fig. 7:**
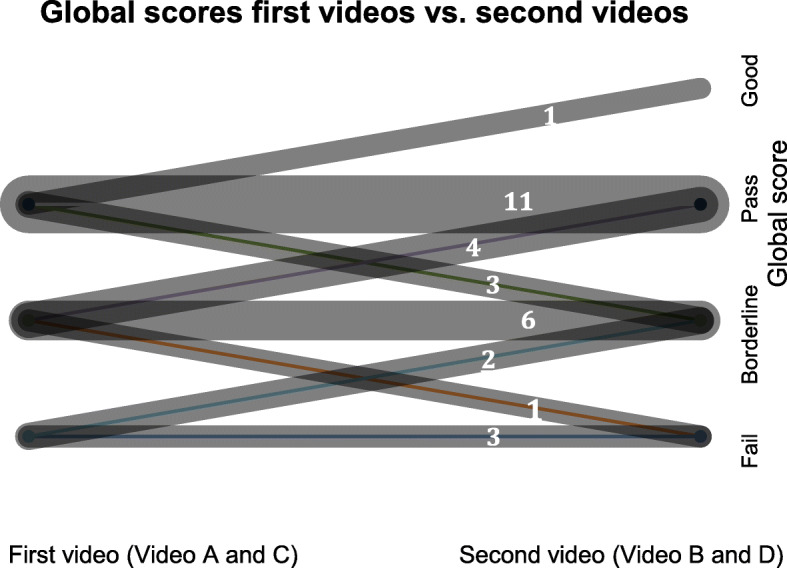
Change in the global scores. Global scores by all examiners in group 1 and 2 are shown (*n* = 31). The points on the left is the global scores given to the first videos A and C (NNEA present) and the points on the right is the global scores given to the second videos B and D (NNEA absent). The thickness of the line and the number on each line represent how many examiners scored in that pattern

The number of the global scores given to videos with and without the NNEA were counted and visualised (Fig. 6). Equal number of ‘Pass’ and ‘Borderline’ grades were given. One and zero ‘Good’ grade was given to videos without the NNEA and videos with the NNEA, respectively. Four and five ‘Fail’ grade was given to videos without the NNEA and videos with the NNEA, respectively. The individual examiner’s change in the global scores given to the first videos A and C (NNEA present) and second videos B and D (NNEA absent) were visualised (Fig. 7). Twenty examiners gave the same scores, seven examiners increased their scores and four examiners decreased their scores.

The Kolmogorov-Smirnova test of normality did not show a normal distribution (*p* < 0.001). Therefore, non-parametric Mann-Whitney test was performed to see the relationship between the accent variable and global scores. The analysis showed the presence of NNEA produced lower global score with the mean rank of 30.58 for videos with the NNEA versus 32.42 for videos without the NNEA. However, this finding was not statistically significant (*p* = 0.661).

## Discussion

This study demonstrates that the NNEA does not affect the checklist items and global scores in OSCEs at a statistically significant level. All analysis indicated that the checklist sum was not affected by the accent variable. Checklists are easier to observe than domain based items and the decisions to award a mark is not influenced by the examiner’s subjective judgment to the same degree as the domain based items or global scales [7]. This could have led to the consistency in the checklist sums when the accent variable was changed. The results also indicated global scores were not influenced by the NNEA. This contradicts studies showing the bias against the NNEAs [24]. It could be considered that the bias against the NNEA was counteracted consciously as a result of examiner training. It is also possible to speculate unconscious mechanisms. When native listeners encountered speakers with NNEAs, they accommodated to the features of the accented speeches [25]. This accommodation could assist examiners to better listen to the student even though the intelligibility was reduced. Another study found that native listeners were likely to conclude that any divergence of speech patterns is because speakers were non-native [26]. This implies that even when a non-native person makes a communication error, it is more likely to be associated with their language background rather than their ability. In this study, examiners could have perceived an unsatisfactory communication as the consequences of the student being non-native.

It is important to note the analysis showed a minor increase in global scores when the NNEA was absent. Although the difference was not statistically significant, the bias against the NNEA could not be excluded from the global scores. There is a suggestion that NNEAs could trigger negative stereotypes [19]. Given the small scale of the study, it would be necessary to carry out further investigation.

Although this study was a small-scale pilot, the findings showed that the examiners were not influenced by the NNEA. This is encouraging for OSCE reliability but also the increasingly diverse medical student population in the UK. Examiners were able to mark based on the student’s performance and not influenced by the protected characteristics. It could be considered that the current OSCE examiner training played a role in ensuring reliability of the assessment. Furthermore, this study has an important implication on current and prospective medical students. Burgess et al. described a ‘stereotype threat’ [27]. When a student recognises the existence of a negative stereotype towards their characteristics, this leads to an unconscious hindrance in the performance. Assurance that students with NNEAs are not subject to disadvantage allow them to perform without the impedance.

The number of the examiner in the study was low due to limited time. The analysis did not demonstrate statistically significant findings. The possibility that this was due to chance would be difficult to exclude considering the small sample size and statistical power. Participants were recruited nationally to ensure they represent the demographics of the UK examiners. However, it is possible that the small cohort produced a different result to the general examiner population. Conducting a similar experiment with more examiners would be important in identifying a reliable relationship between NNEAs and OSCE marks.

Although the examiners were blinded from the study aim, some might have deduced it during the marking. There were only two videos for each examiner. It would be possible for them to notice the speeches in the videos were different. This could have produced bias as more conscious effort would be taken to minimise the marking variation. Using more videos with variations in accents, student characteristics and scripts would improve the blinding.

As shown in the result, the pattern of the score change for checklist sums was different for group 1 and group 2. It could be argued that the order the examiners watched scripts 1 and 2 influenced how the performance was perceived. This might have been a con-founding variable. Such effect could also be reduced with the use of more video variations. This was not feasible in this study due to resource constraints. Examiners could further be asked after the submission of the marks to comment on what they thought the study was investigating. This information would aid in evaluating the validity of the study. Moreover, requesting their feedback on the degree of control between two videos and the quality of the recordings would have been useful in establishing the reliability of the result.

Only one actor and one OSCE scenario material was available due to resource restriction. Protected characteristics of the actor such as gender and ethnicity could possibly impact the examiners’ judgements. Moreover, evidence suggests that perceived stereotypes could be markedly different depending on the type of the accent [22]. Using several actors with different characteristics and accents would improve the reliability of the study. Only one OSCE scenario was viewed in the experiment but in reality, the students’ outcomes are determined by their performances across several stations. Therefore, use of several scenarios would lead to higher validity. As the context of the OSCE station varies according to the level of students, it is important to recognize this study only looked at a scenario written for clinical year medical students and may not be applicable for non-clinical year students or postgraduate applicants.

In this study, the language background of the examiner was not asked. This information would be valuable because the native/non-native status is highly relevant to how people assess NNEAs since stereotyping is dependent on the individual’s social identity [28]. How the communication divergence is evaluated is also different depending on the native/non-native status of a listener [26]. Thus, the result could be influenced by the language status of the participants.

It was not possible to look at the effect of the demographics information in this study since two markings from one examiner were used in the analysis. It would be possible to analyse the demographics impact by grouping the data based on smaller subsections of the participant group. This would allow demographic information and accent variables to be treated as isolated variables. More examiners are required to conduct this analysis reliably.

The validity of the result could be impacted by the experimental setting. In this study, examiners were not subjected to time restrictions in awarding the marks. They would be given a few minutes to complete marking each student in reality. Additionally, they were observing the student online in a recorded video using their own computers. In a real OSCE, they would be allocated a spot in a station to observe the student directly. The removal of the potential stressors encountered in an actual OSCE could possibly lead to variances in the examiners’ judgements.

It would also be valuable to investigate the effect on the simulated patient evaluation. The mark scheme used in the experiment had one item for empathy which the simulated patient was asked to contribute to. Analysis of this item would explore the influence of NNEAs on the assessment marks comprehensively. It would also show the influence of accents on the wider public. The patient evaluation on clinical communication is affected by the patient’s language background [26]. It would be valuable to explore how the patient evaluation changes due to the accent.

A quantitative study alone would not provide an insight into why the NNEAs might be influencing the examiners. Study methods such as interviews should be considered to explore this issue further. Qualitative studies may reveal how NNEAs could be interpreted in light of clinical competence. It would be beneficial to discuss how NNEAs in the clinical context are viewed by all stakeholders. This would inform the training process of OSCE examiners the future.

## Conclusion

In this study, the NNEAs were shown to have little influence on OSCE examiners. It highlights an important topic for current medical education in the UK, given the increasingly diverse population. The findings of this study would provide a starting point for further investigation. The findings in this study have shown that there was no bias against a student with a NNEA. It is important to acknowledge that this was a pilot study. Considering the small sample size and influences of uninvestigated examiner characteristics, further research is required to confirm the findings.

## Supplementary information

**Additional file 1.** CONSORT 2010 checklist of information to include when reporting a randomised trial.

## Data Availability

The datasets used and/or analysed during the current study are available from the corresponding author on reasonable request.

## Abbreviations

GMC: General Medical Council
NEA: Native English Accent
NNEA: Non-native English Accent
OSCE: Objective Structured Clinical Examination
QMPlus: Queen Mary Plus

## References

[1] Norman G (2002). Research in medical education: three decades of progress. BMJ..

[2] Epstein RM (2007). Assessment in medical education. N Engl J Med.

[3] Gormley G (2011). Summative OSCEs in undergraduate medical education. Ulster Med J.

[4] Carraccio C, Englander R (2000). The objective structured clinical examination: a step in the direction of competency-based evaluation. Archives of pediatrics & adolescent medicine.

[5] General Medical Council. Assessment in undergraduate medical education: advice supplementary to Tomorrow’s Doctors. London: General Medical Council; 2009. https://www.gmc-uk.org//media/documents/Assessment_in_undergraduate_medical_education___gu idance_0815.pdf_56439668.pdf. Accessed 29 Apr 2019.

[6] Khan KZ, Gaunt K, Ramachandran S, Pushkar P (2013). The objective structured clinical examination (OSCE): AMEE guide no. 81. Part II: Organisation & Administration. Med Teach.

[7] Brannick MT, HT EK, Prewett M (2011). A systematic review of the reliability of objective structured clinical examination score. Med Educ.

[8] Schwartzman E, Hsu DI, Law AV, Chung EP (2011). Assessment of patient communication skills during OSCE: examining effectiveness of a training program in minimizing inter-grader variability. Patient Educ Couns.

[9] Haq I, Higham J, Morris R, Dacre J (2005). Effect of ethnicity and gender on performance in undergraduate medical examinations. Med Educ.

[10] Woolf K, Potts HW, McManus IC (2011). Ethnicity and academic performance in UK trained doctors and medical students: systematic review and meta-analysis. Bmj..

[11] Wass V, Roberts C, Hoogenboom R, Jones R, Van der Vleuten C (2003). Effect of ethnicity on performance in a final objective structured clinical examination: qualitative and quantitative study. BMJ..

[12] SchoonheimKlein M, Hoogstraten J, LLMH H, Aartman I, Van der Vleuten C, Manogue M, Van der Velden U (2007). Language background and OSCE performance: a study of potential bias. Eur J Dent Educ.

[13] Liddell MJ, Koritsas S (2004). Effect of medical students’ ethnicity on their attitudes towards consultation skills and final year examination performance. Med Educ.

[14] Mann C, Canny B, Lindley J, Rajan R (2010). The influence of language family on academic performance in year 1 and 2 MBBS students. Med Educ.

[15] Wass V, Roberts C, Hoogenboom R, Jones R, Van der Vleuten C. Effect of ethnicity on performance in a final objective structured clinical examination: qualitative and quantitative study. BMJ. 2003;326(7393):800–3. 10.1136/bmj.326.7393.800 PMC153100 12689978

[16] Munro MJ (2003). A primer on accent discrimination in the Canadian context. TESL Canada Journal.

[17] Major RC (2010). First language attrition in foreign accent perception. Int J Biling.

[18] De Jong KJ (2018). Sensitivity to foreign accent. Acoust Today.

[19] Munro MJ, Derwing TM, Morton SL (2006). The mutual intelligibility of L2 speech. Stud Second Lang Acquis.

[20] Pantos AJ, Perkins AW (2013). Measuring implicit and explicit attitudes toward foreign accented speech. J Lang Soc Psychol.

[21] Deprez-Sims AS, Morris SB (2010). Accents in the workplace: their effects during a job interview. Int J Psychol.

[22] Carlson HK, McHenry MA (2006). Effect of accent and dialect on employability. J Employ Couns.

[23] Louis WR, Lalonde RN, Esses VM (2010). Bias against foreign born or foreign trained doctors: experimental evidence. Med Educ.

[24] Cargile AC (2002). Speaker evaluation measures of language attitudes: evidence of information-processing effects. Lang Aware.

[25] Munro MJ, Derwing TM (2001). Modeling perceptions of the accentedness and comprehensibility of L2 speech: the role of speaking rate. Stud Second Lang Acquis.

[26] Alexander ST (2001). Sincerity, intonation, and apologies: a case study of ai EFL and ESL learners. [dissertation].

[27] Burgess DJ, Warren J, Phelan S, Dovidio J, Van Ryn M (2010). Stereotype threat and health disparities: what medical educators and future physicians need to know. J Gen Intern Med.

[28] Hornsey MJ (2008). Social identity theory and self-categorization theory: a historical review. Soc Personal Psychol Compass.

